# Clinical Society of New York Polyclinic Medical School and Hospital

**Published:** 1911-12

**Authors:** 


Reported for Texas Medical Journal.

### Clinical Society of New York Polyclinic Medical School and Hospital.

Meeting November 6, 1911.

Dr. Packard presented a case of caisson disease simulating locomotor ataxia.

The patient, a man 55 years of age, came complaining of chronic rheumatism. His history was unique—was perfectly well until ten years ago, when he began to work in the tunnel. This resulted in an attack of caisson disease, with lightning pains in the right leg and thigh. There were no other symptoms, and after recovery he returned to the caisson and continued to work until a month ago. During his work in the tunnel he had four or five attacks.

Examination showed him to be suffering from a peculiar type of caisson disease, which resembled locomotor ataxia. The symptoms were Argyle-Robinson pupil, Romberg's sign, lightning pains in right leg, some pain in left, and an osteo-arthritis of the knee.

The question arose as to whether he was suffering from syphilis or caisson disease. Wassermann and Noguchi tests were negative, as were injection tests of the spirochaeta pallida. He denied all history of syphilis.

Dr. Beal said that in his opinion not all cases of locomotor ataxia were due to syphilis. This case had reacted negatively to Wassermann, Noguchi, and to the spirochaeta pallida test. From the examination it looked like tabes.

Dr. Gilday thought that 60 per cent was low for specific tabes cases. His own experience had shown a larger percentage. In regard to the value of the Wassermann or Noguchi reaction, preceding or in the stage of tabes, he had had negative reactions despite a positive history of syphilis. He felt that in post-syphilitic cases these reactions were not reliable.

Dr. Gilday inquired whether in post-syphilitic cases the spirochaeta pallida had produced a degeneration in the spinal cord, and if so, would the dead spirochaetae give a reaction?

Dr. Packard replied that the spirochaetae were not dead, but dormant, and this condition was what made the injection of "606" effective.

Dr. Sinclair asked if the patient had been drinking any alcohol before the tests were made, as it is well known that if a man drinks a glass or two of whisky before the Wassermann or Noguchi tests are made, they are apt to give negative results. Even one glass of whisky will effect the test.

Case for differential diagnosis between cirrhosis of the liver and carcinoma:

Dr. Beal presented this case for diagnosis, because he wished to differentiate between enlargement of the spleen, in cirrhosis, and absence of enlargement of the spleen in carcinoma.

In his opinion, with cirrhosis of the liver, there is always enlargement of the spleen, and with carcinoma never enlargement of the spleen.

The patient shown was perfectly well up to May last, when he commenced to fail. In June he fell sick, and since then has lost half his weight. He has been ascitic, and been tapped once, and sixty to seventy ounces of fluid removed. The spleen did not seem particularly enlarged, but just over the pyloris was a hard carcinomatous mass. The man had the carcinoma symptoms, but no pain.

Dr. Packard asked if there would not be bloody fluid in carcinoma. Dr. Beal replied that bloody fluid was not necessarily present. Dr. Packard said that in cirrhosis of the liver blood tinged fluid was rare, unless it was due to the rupture of some small vessel made by the surgeon in entering the abdomen, and so causing a return flow which was blood tinged. In all cases of cirrhosis of the liver there is enlargement of the spleen, because the portal circulation is obstructed. This case might be caused by a cirrhosis of the liver or carcinoma of the liver. It seemed to him since there was portal obstruction cirrhosis might have large spleen. He believed that carcinoma did have an enlarged spleen.

Dr. Strauss thought that care should be exercised in eliminating other diseases of the liver, which might simulate carcinoma and cirrhosis in the case observed. Enlarged liver with nodules has shown tubercular abscess on operation.

Dr. Gilday suggested that an exploratory incision might have solved the diagnosis, and it would do no more injury than tapping.

Dr. Tovey asked if Dr. Beal had made a rectal examination, as the Mayos have found metastasis of the rectum in carcinoma of the liver.

Dr. Reich said that malignancy was usually found with bloody fluid.

Dr. Beal said that in his experience the fluid in cirrhosis was of the same character as that in malignancy. He said he was glad that most of those present found no enlargement of the spleen, as he wished to bring out its normal size in carcinoma.

Two cases showing results of Whitehead operation for hemorrhoids, presented by Dr. Dryfus:

The first patient came complaining of severe itching and some granulation at the rectum. He had been operated on during the summer by a modification of the Ball operation, but was still complaining of several points of itching. He was in bed for a week or so, with loss of control, which subsequently had been restored.

The other case was operated upon in England six months ago by the regular Whitehead method. This patient came under observation complaining of itching, discharge, pain, with slight blood in the stools. Examination showed what looked like ulcer, but proved to be mucous membrane. Too much having been cut off, it had not healed, and the man was continually irritated. The Ball modification of the Whitehead was very successful. Many of the supposed strictures of the rectum were due, after a regular Whitehead operation, to the sloughing of the mucous membrane. In a primary union this symptom is obviated.

Dr. Lynch said that these two cases had been shown because the one operated upon by the Whitehead method had been told that nothing could be done for him, while as a matter of fact, he was completely relieved by a simple plastic operation.

